# Application of network pharmacology in the study of mechanism of Chinese medicine in the treatment of ulcerative colitis: A review

**DOI:** 10.3389/fbinf.2022.928116

**Published:** 2022-09-27

**Authors:** Shihao Zheng, Tianyu Xue, Bin Wang, Haolin Guo, Qiquan Liu

**Affiliations:** ^1^ Graduate School, Hebei University of Traditional Chinese Medicine, Shijiazhuang, China; ^2^ Department of Spleen and Stomach, First Affiliated Hospital of Hebei University of Traditional Chinese Medicine, Shijiazhuang, China

**Keywords:** network pharmacology, ulcerative colitis, mechanism of action, traditional Chinese medicine pharmacology, a review

## Abstract

Network pharmacology is a research method based on a multidisciplinary holistic analysis of biological systems, which coincides with the idea of the holistic view of traditional Chinese medicine. In this review, we summarized the use of network pharmacology technology through studying Chinese medicine single medicine or Chinese medicine compound research ideas and methods for the treatment of ulcerative colitis, based on the application of the current network pharmacology in Chinese medicine research, including the important role in the mechanism of the prediction and verification, to search for new ideas for disease diagnosis and treatment, this study summarizes the application of network pharmacology in the treatment of ulcerative colitis in traditional Chinese medicine, including monotherapy and compound therapy, and considers that relevant research studies have fully demonstrated the function characteristics of the multi-component, multi-target, and multi-pathway of traditional Chinese medicine, and can also explain the connotation of “selecting appropriate treatment methods according to the differences and similarities of pathogenesis” of traditional Chinese medicine. Finally, we raised important questions about the prospects and limitations of network pharmacology, such as differences caused by different data collection methods, a considerable lag, and so on.

## 1 Introduction

Network pharmacology, as an emerging discipline, was first proposed by Hopkins, a British pharmacologist, in Nature Biotechnology in 2007. Under the premise of the rapid development of network databases, it is based on the theories of pharmacology, bioinformatics, and other disciplines. Further use of visualization technology, high-throughput technology, network analysis, and other methods to explore the single drug or compound for the treatment of diseases of TCM effective mechanism, and from the perspective of macro expound Chinese medicine single medicine or Chinese medicine compound and the interaction mechanism between the disease, reflects many components, the characteristics of targets and pathways (Hopkins, 2007; Li and Zhang, 2013; Wu et al., 2016).

Network pharmacology is combined with systematic biomedical technology. Through computer software and the TCM database website, the unique advantages of TCM in treating diseases are combined with single drugs or compound drugs of traditional Chinese medicine. Various active ingredients of single drugs or compound drugs of traditional Chinese medicine are analyzed by the network pharmacology method, and the key action mechanism of treating diseases is discussed. It provides a better reference for the treatment of diseases and the invention of clinical new drugs. The network theory of “drug–target–gene–disease” in network pharmacology and its holistic analysis of biological systems coincide with the holistic concept proposed by traditional Chinese medicines (Hopkins, 2008). Nowadays, compared with traditional pharmacology, the application scope of network pharmacology is expanding day by day, which can effectively explore the specific mechanism of TCM in disease treatment, study the application scope of TCM in disease treatment, and better reflect the rationality of TCM theory (Liu et al., 2017; Zhan and Su, 2018; Guo et al., 2019; Han et al., 2019). Network pharmacology, as a new discipline that selects specific signaling nodes for multi-target drug molecular design, emphasizes the multi-way regulation of signaling pathways, improves the therapeutic effects of drugs, reduces toxic and side effects, and thus improves the success rate of clinical trials of new drugs. This study reviews the current research on the mechanism of Chinese medicine in the treatment of ulcerative colitis based on network pharmacology, in order to find the problems existing in relevant research fields and expand ideas for future research.

As a chronic immune-mediated inflammatory disease of the colon, the development of ulcerative colitis (UC) is thought to be associated with an inappropriate immune response to gut commensal microbes (Abraham and Kane, 2012; Ng et al., 2013). Epidemiology shows that the most common age of ulcerative colitis is in the second to fourth decade of life (Cosnes et al., 2011; Molodecky et al., 2012). Today, the incidence of UC is rising rapidly compared with before, and such diseases cover a wide range of people and can occur at any age. UC has many complex conditions in clinical management, and there are many complications, and there is no clear treatment method (Ng et al., 2017).

**FIGURE 1:**
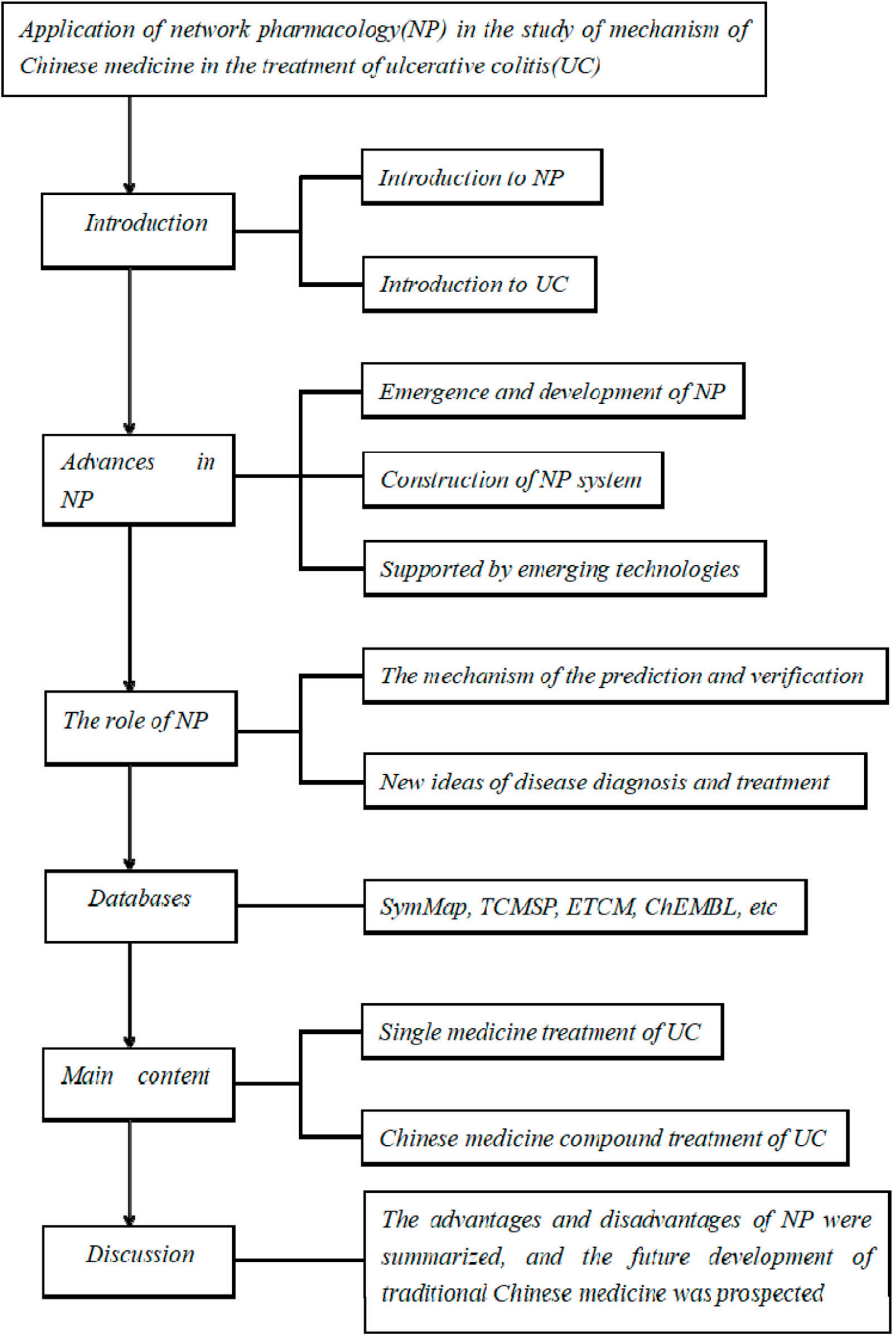
Framework figure.

During the treatment of UC, multiple steps are involved, such as the recovery of epithelial cells and the repair of the damaged intestinal mucosa, which can eventually return to normal (Neurath and Leppkes, 2019). The conventional treatment of UC in modern medicine includes drugs such as mesalazine or glucocorticoids, and some patients will get effective relief, but the side effects of these drugs are very obvious, and it is not suitable for long-term excessive use (Wan et al., 2014). However, in recent years, Chinese medicine has played a great role in the treatment of ulcerative colitis. More and more people have further explored the treatment of such inflammatory bowel diseases from the perspective of traditional Chinese medicine and thus achieved greater gains. The framework figure of the study is shown in Figure 1.

## 2 Advances in network pharmacology

### 2.1 Emergence and development of network pharmacology

Nowadays, while network pharmacology technology is applied by many scholars in the medical field, we should see that the rise of network pharmacology technology is not accidental. It is well known that bioinformatics and computational biology have laid the foundation for the emergence and development of network pharmacology.

Bioinformatics is an interdisciplinary subject that integrates mathematics, biology, and computer science. It includes the collection, processing, storage, distribution, analysis, and interpretation of biological information data. Today, we can apply ideas and methods from mathematics and computer science combined with biological tools to elucidate and understand the biological meaning contained in large amounts of data. Computational biology also belongs to a branch of biology. It mainly builds statistical and mathematical models for biological phenomena, analyzes a large number of omics data, and extracts biologically meaningful rules. There is no doubt that it is becoming the core content of modern biological research. It is the progress in these fields that provides the prerequisites for the occurrence and development of network pharmacology, and the holistic view of traditional Chinese medicine coincides with the new generation of medical research models characterized by systems and networks. In the field of traditional Chinese medicine, a new method characterized by a systematic and overall “network target” has emerged, and a core method and theory of network pharmacology have emerged as the times require (Wang X. et al., 2021).

### 2.2 Construction of the network pharmacology system

To construct the network pharmacology system, we should first understand the internal relationship of the “drug–target–gene–disease” network, and reveal or predict the mechanism of action of single or compound Chinese medicine on the disease through the network relationship. On the basis of consulting a large number of literature works, the database was searched in depth to obtain the chemical components contained in the single medicine or compound medicine of traditional Chinese medicine, and the corresponding action targets of the chemical components were further obtained by searching the database. After obtaining the composition and target of the drug, the gene target of the disease was obtained by referring to the literature retrieval database again. Through further integration and analysis, the relationship network of “drug–target–gene–disease” was finally obtained, and the network pharmacology system corresponding to the treatment of the disease was successfully constructed. Network pharmacology through the construction of the biological network, screens out the key targets and related pathways of TCM components in the treatment of diseases, so as to clarify the pharmacologic basis of drugs, further clarify the different aspects of TCM treatment of diseases in the way of multi-components, multi-targets, and multi-pathways, and have a synergistic effect on each other (Qi et al., 2016).

### 2.3 Supported by emerging technologies

Supported by emerging technologies such as molecular interaction technology, high-throughput technology, and omics technology, network pharmacology further improves and validates the constructed “drug–target–gene–disease” biological network, and improves the accuracy of prediction models. Molecular interaction technology, as one of the important emerging technologies, mainly includes biofilm interference technology, plasma resonance technology, and nano-liquid chromatography–mass spectrometry analysis technology, which can quickly reflect the interaction between receptor proteins and drug molecules (Hsieh and Korfmacher, 2006; Guo, 2012). In the process of experiments, the use of high-throughput technology can realize automatic operation, batch, precise processing of cell experiments produced by the sample, and can quickly obtain a lot of useful information. Some studies have confirmed that network pharmacology is more effective in revealing the principle of small molecule regulation by using high-throughput technology when constructing a “complex protein–disease” network (Zhang et al., 2019a). The last omics technology mainly includes genomics, proteomics, and metabolomics, which can more intuitively observe the different regulatory effects of single or compound Chinese medicine at different levels. As an emerging scientific technique, proteomics can effectively reveal potential target proteins or protein biomarkers, thus transforming the promises of TCM into powerful modern therapies (Suo et al., 2016). These emerging technologies have played a strong supporting role in the construction of the biological network, transforming the complex connections and complex information inside the network into a more intuitive visual network, reducing the difficulty of text information, so that readers can further study and research.

### 2.4 The role of network pharmacology in the development of Chinese medicine

#### 2.4.1 The mechanism of the prediction and verification

Prediction of a single drug or compound Chinese medicine targets for the disease and its mechanism of action is also the process of constructing the “drug–target–gene–disease” network, the process of the successful prediction of a drug’s active ingredient, after target gene and the related signal pathways, and related animal experiment or cell to the next step, the predicted results confirmed to make it more persuasive. It can better guide the clinical use of targeted single Chinese medicine to treat diseases, or determine the importance of each Chinese medicine in the compound according to the number of target genes that each Chinese medicine acts on diseases, better use the principle of drug compatibility, and divide the monarch, minister, helper, and envoy, so as to maximize the curative effect of drugs.

#### 2.4.2 New ideas for disease diagnosis and treatment

There is such a principle in the diagnosis and treatment of diseases in traditional Chinese medicine: if the pathogenesis is the same, the law of treatment is the same. Different pathogenesis, different treatment principles, this kind of treatment method and modern medicine precision treatment happens to coincide, it can be seen that the importance of identifying the pathogenesis in the process of disease treatment. Analysis of different types by using the method of network pharmacology has the same disease pathogenesis, for two or more diseases of the connection between the pathogenesis and disease, and the rare one is established by the use of the network pharmacology method, refractory diseases of target genes, and related signaling pathways, combined with previous experience to select the suitable drugs, can achieve twice the result with half the effort. It accords with the idea of combining disease and pathogenesis in traditional Chinese medicine and provides a new guidance for the treatment of complicated and complicated diseases in clinic.

## 3 Network pharmacology common database

### 3.1 SymMap database

The SymMap (https://www.symmap.org/) database (Wu et al., 2019) is a TCM syndrome association database, which includes 499 herbal medicines registered in the Chinese Pharmacopoeia and corresponding 1717 TCM symptoms. The symptoms are precisely matched to 961 medical terms used in modern medicine. In addition, the SymMap database also includes 5,235 diseases associated with these syndromes, 4,302 drug targets, 19,595 herbal components, and the associations between these types of data. In this way, the SymMap database links traditional Chinese medicine and modern medicine from the phenotypic to the molecular level.

### 3.2 Traditional Chinese medicine systems pharmacology database and analysis platform

TCMSP (traditional Chinese medicine systems pharmacology database and analysis platform, http://tcmspw.com/tcmsp.php) (Ru et al., 2014) contains 499 Chinese medicines registered in the Chinese Pharmacopoeia, and also includes 29,384 components, 3,311 targets, and 837 related diseases. The database uses the HIT database prediction algorithm to obtain effective relationships between drugs and targets. The disease information in this database comes from the TTD database and the PharmGKB database. We can search and screen Chinese medicine single drug or compound prescriptions in this database. It is a common database for screening Chinese medicine ingredients and targets.

### 3.3 Encyclopedia of traditional Chinese medicine database

The ETCM (encyclopedia of traditional Chinese medicine, http://www.tcmip.cn/ETCM/index.php/Home/) database (Xu et al., 2019) is a comprehensive resource database of traditional Chinese medicine, which contains information on 402 herbs, 3,959 traditional Chinese medicine compounds, 7,284 traditional Chinese medicine chemical components, 2,266 drug targets, and 4,323 related diseases. The herbal medicine contains specific information such as meridian, medicinal properties, medicinal taste, ingredients, indications, quality control standards, and origin; the compound formula contains information such as name, composition, dosage form, ingredients, and indications; the herbal ingredient contains the molecular formula of the compound, a variety of physical and chemical indicators, molecular weight, drug-like properties, ADME parameters, and other information. It can also perform functional enrichment analysis on target gene information, which is a very practical database.

### 3.4 ChEMBL database

The ChEMBL (https://www.ebi.ac.uk/chembl) database (Gaulton et al., 2012) was established by the European Molecular Biology Laboratory (EMBL) to provide the scientific community with comprehensive information on a variety of biologically active compounds with drug targets for basic research through a range of tools and services. In addition to extracting data from the medicinal chemistry literature, this database also adds new sources of bioactivity data to the database, including disposition data and drug metabolism in patents, crop protection data, and bioactivity data.

### 3.5 Search tool for interactions of chemical database

The STITCH (search tool for interactions of chemical, http://stitch.embl.de/) database (Kuhn et al., 2008) is a database of predicted interactions between proteins and chemical drugs, their interactions include direct or indirect associations, data derived from knowledge transfer between organisms or derived from computational predictions, and interactions from other databases such as OMIM, PubMed, and BioGRID, which currently contain 9,643,763 proteins from 2,031 organisms.

### 3.6 STRING database

The STRING (https://string-db.org/) database (Snel et al., 2000) provides key assessments and integrations of protein–protein interactions, including physical and functional associations. This database lays the foundation for many functional synergistic relationships and interactions among proteins and enriches their relevant background in systems biology (Table 1).

**TABLE 1:** Public databases related to TCM network pharmacology.

Type		Name	Description	Website for database or tool	Reference
Databases	TCM-related databases	SymMap	TCM syndrome association database linking TCM and modern medicine from phenotype to molecular level	https://www.symmap.org/	Wu et al. (2019)
		TCMSP	Database and analysis platform that can retrieve information on key components and targets of traditional Chinese medicine	http://tcmspw.com/tcmsp.php	Ru et al. (2014)
		ETCM	Comprehensive database of traditional Chinese medicine resources	http://www.tcmip.cn/ETCM/index.php/Home/	Xu et al. (2019)
	Drug-related databases	ChEMBL	Open database containing rich ADME information, functions of biologically active compounds etc.	https://www.ebi.ac.uk/chembl	Gaulton et al. (2012)
		STITCH	Database of predicted protein and chemical interactions	http://stitch.embl.de/	Kuhn et al. (2008)
	Target-related databases	STRING	Database of known or predicted protein–protein interactions	https://string-db.org/	Snel et al. (2000)

## 4 Application of network pharmacology in the treatment of ulcerative colitis with traditional Chinese medicine

The diagnosis and treatment of diseases in traditional Chinese medicine often proceed from the whole, and different treatment methods are adopted according to the different pathogenesis. Network pharmacology is the same. First, the relationship network of “drug–target–gene–disease” is constructed from the whole point of view, and then the target and pathway of TCM single drugs or compound drugs are further explained to treat diseases, and the specific mechanism of action is elaborated. Although some studies or mechanisms of ulcerative colitis in the world have been confirmed by clinical trials, few researchers can fully and thoroughly elaborate on the specific mechanism of action, so it is necessary to explore the specific mechanism of action of traditional Chinese medicine on ulcerative colitis by using the network pharmacology method. This study reviews the research articles on the treatment of ulcerative colitis with traditional Chinese medicine by network pharmacology in recent 10 years, and divides them into monotherapy and compound studies according to the different numbers of drugs studied (Table 2).

**TABLE 2:** Network pharmacology analysis of traditional Chinese medicine in the treatment of ulcerative colitis.

Reference	Formula and herb	Object	Main ingredient	Main target	Main signaling pathway	Outcome
Liu S. et al. (2021)	Curcuma	Molecular docking	Campesterol, CLR, stigmasterol	AKT1, EGFR, STAT3	PI3K-Akt, JAK-STAT, MAPK	Alleviate the pathological manifestations of UC and reduce the expression of TNF-α and STAT3
Liu B. et al. (2021)	Caulis Sargentodoxae	None	Physcion, emodin, meso-dihydroguaiaretic acid	SRC, PIK3CA, MAPK1	HIF-1, PI3K-Akt	By regulating the HIF-1 signaling pathway and the PI3K-Akt signaling pathway, the 8 components play a synergistic role
Chen et al. (2020)	*Sophora flavescens* (kushen)	Human	Quercetin, luteolin, matrine	IL-6, MYC, CCND1	PI3K-Akt	Kushen-based TCM formulations for the treatment of UC showed a significantly higher clinical remission rate and lower incidence of adverse events
Zhang Y. et al. (2019)	*Paeonia lactiflora*	None	Kaempferol, beta-sitosterol, (+)-catechin	IL-6, BCL2, AKT1	Pathways in cancer, TNF, Hepatitis B	*Paeonia lactiflora* exerts its therapeutic effect on UC through multiple targets and multiple pathways, but it lacks experimental verification
Zhang et al. (2021b)	*Schisandra chinensis*	C57BL/6 mice	Schisandrin B	IL-1β, IL-6, IL-18	AMPK/Nrf2	Schisandrin B reduces the epithelial cell injury of colitis through regulating pyroptosis by AMPK/Nrf2/NLRP3 inflammasome
Cui et al. (2020)	*Quercus infectoria*	Male BALB/c mice	Gallincin	IL-1β, TNF-α, p-ERK1/2	Ammonium ion, Immune	Gallincin modulates colonic barrier function and reduces colitis-related inflammation
Zhou Q. et al. (2021)	Coix seed feed	Eight-week-old female Kunming mice	Crude protein, crude fat, crude fiber	T-cell regulation	T-cell receptor, TNF	Coix seed feed can alleviate oxidative stress, decrease inflammatory cytokine secretion and pathological score
Gu et al. (2020a)	Indigo naturalis	Molecular docking	Qingdainone, isovitexin, isoindigo	IL-6, TNF, TP53	Jak-STAT, IL-17, toll-like receptor	Therapeutic effect of Indigo naturalis on UC may involve activation of systemic immunity
Niu et al. (2021)	SCDP	Molecular docking	Quercetin, wogonin, beta-sitosterol	AKT1, JUN, TNF	P53, PI3K-Akt, IL-17	Key active ingredients of SCDP play a therapeutic role in UC by mainly intervening with the key signaling targets, such as the inflammatory response and oxidative stress
Zhang et al. (2021a)	GZW	Normal mouse RAW264.7 cells, SD rats	Quercetin, kaempferol, beta-sitosterol	JUN, STAT1, RELA	NF-kB, STAT3, IL-6	GZW effectively relieves UC symptoms through related pathways such as immunity, inflammation, and oxidative stress
Chen et al. (2021b)	HTD	Female Swiss mice	Wogonin, glycyrrhizic acid, liquiritigenin	IL-1β, IL-6, MMP1	Pathways in cancer, IL-17	Pathological conditions of colon tissues of the mice were improved compared with those in model group
Wei et al. (2021)	ZJP	None	Berberine, obacunone, quercetin	JUN, MAPK1, PIK3CA	Toll-like receptor, MAPK, PI3K-Akt	ZJP can regulate intestinal inflammation and maintain the integrity of the intestinal mucosal epithelial barrier
Yu et al. (2021)	HYJJ	C57BL/6 mice	Hederagenin, karanjin, beta-sitosterol	IL-2, IL-10, IL-12	TNF, toll-like receptor, Nod-like receptor	HYJJ re-establishes homeostasis of the gut epithelium during colitis by suppressing inflammation and orchestrating cytokines interaction
Liu et al. (2021b)	XLP	SD rats	Coptisine, berberine, magnoflorine	JAk2, STAT3, HIF-1α	Th17 cell differentiation, Jak-Stat, PI3K-Akt	Apharmacokinetic study revealed that the nine ingredients of XLP are exposed in the plasma and colon tissue, which demonstrates its pharmacological effect on UC
Wang et al. (2021a)	XJD	Molecular docking	β-sitosterol, kaempferol, formononetin	ESR1, JUN, MAPK1	Endocrine resistance, breast cancer, MAPK	Active ingredients of XJD may regulate the inflammatory and oxidative stress–related pathways of colon cells during the course of UC by binding to the hub gene targets
Yang et al. (2019)	Quyushengxin formula	None	Quercetin, ferulic acid, palmatine	ESR1, PTGS2, NOS2	mTOR, T-cell receptor, JAK-STAT	Quyushengxin may act on immune and inflammation-related targets to suppress UC progression in a synergistic and additive manner
Chen W. et al. (2021)	HHS	BALB/c mice	Kaempferol, luteolin, nobiletin	EGFR, KDR, PIK3R1	HIF-1, PI3K/AKT, VEGF	HHS in the body has been proven to be effective in alleviating UC symptoms
Liu J. et al. (2021)	HHS	Mouse mononuclear macrophage leukemia cells (RAW264.7)	Quercetin, luteolin, nobiletin	JUN, TP53, ESR1	PI3K-AKT	Quercetin could inhibit phosphorylated c-Jun, the levels of inflammatory factors and PI3K-Akt signaling pathway in LPS-induced RAW264.7 cells
Wei et al. (2020)	GGQL	Molecular docking	Quercetin, stigmasterol, kaempferol	PPARG, AR, RELA	Toll-like receptor, IL-17, Tumor necrosis factor	GGQL can promote intestinal recovery by anti-inflammatory, antioxidant, inhibiting tumor gene transcription etc.
Xu et al. (2021)	GGQLD	Molecular docking	Quercetin, kaempferol, wogonin	IL-6, CXCL8, CCL2	IL-17, Th17 cell differentiation	GGQLD can modulate the immune system to promote remission in UC
Yu et al. (2020)	Banxia xiexin decoction	None	5-Hydroxytryptamine synaptic, arachidonic acid	ALB, IL-6, CEGFA	HIF-1, NF-kB	Banxia xiexin decoction can have the same therapeutic effect on two different diseases, UC and depression, through inflammatory response, oxidative stress response etc.
Huang et al. (2021)	WMP	Molecular docking	Quercetin, kaempferol, ginsenoside rh2	CCL2, HIF1-A, JUN	IL-17, TNF, AGE-RAGE	Mechanisms of action of WMP involve modulation of immunity, anti-oxidation, and anti-inflammation
Ji et al. (2020)	SNT	SD rats	Beta-sitosterol, sitosterol	CRP, COL12A1, MPO	ECM–receptor interaction, PI3K-AKT, PPAR	SNT can reduce the colonic mucosal injury index and disease activity index
Wen et al. (2021)	JQP	C57BL/6 wild-type (WT) male mice	Quercetin, beta-sitosterol, stigmasterol	IL-6, JUN, TP53	NF-kB, HIF-1, TNF	JQP regulates the Th17/Treg cell balance in DSS-induced mice
Zhou C. et al. (2021)	GJHQHLRSD	Molecular docking	Kaempferol, worenine, palmidin A	BCL2L1, NR3C1, ALOX5	EGFR, TNF, JAK-STAT	GJHQHLRSD acts by modulating EGFR signaling in UC therapy
Zheng et al. (2021)	JPQCHSR	Molecular docking	Quercetin, luteolin, kaempferol	STAT3, AKT1, TP53	AGE-RAGE, IL-17, TNF	JPQCHSR can play an important role in repairing intestinal immune damage and reducing the expression of inflammatory factors
Ding et al. (2021)	HDC	Male C57BL/6 mice	Polydatin, gallic acid, quercetin	IL-6, TP53, PTGS2	JAK2/STAT3, IL-17	HDC can downregulate the secretion of pro-inflammatory cytokines during UC treatment
Gu et al. (2020b)	FSEC	None	Quercetin, kaempferol, luteolin	IL-6, VEGFA, PTGS2	IL-17, HIF-1, TNF	FSEC plays a comprehensive role in the treatment of UC, such as immune recognition, anti-inflammatory, and antioxidant

### 4.1 Single medicine treatment of ulcerative colitis

One study found that 12 active ingredients in Curcuma, including 148 target genes, were screened out by searching the database, and 54 potential targets for the treatment of ulcerative colitis were screened out by using molecular docking technology. A total of 24 core proteins were screened out by molecular docking technology. These targeted proteins effectively treat and relieve ulcerative colitis through the PI3K-Akt signaling pathway, JAK-STAT signaling pathway, and MAPK signaling pathway, to achieve the purpose of treating the disease (Liu et al., 2021d). In the study of the molecular mechanism of the therapeutic effect of Caulis Sargentodoxae on ulcerative colitis, eight active components play a significant role and involve 102 key targets, which are closely related to the positive regulation of cell vitality, reaction to toxic substances, leukocyte migration, cell response to drugs, and inflammatory response. It was confirmed that the PI3K-Akt signaling pathway and HIF-1 signaling pathway are key targets of the UC-related signaling pathway (Liu et al., 2021a). *Sophora flavescens* has a certain effect on ulcerative colitis. Some studies have constructed a composition–target–pathway network model of *Sophora flavescens*, including five main components including quercetin, and involving three main targets such as IL-6, which play an effective role by acting on the PI3K-Akt signaling pathway (Chen et al., 2020). *Paeonia lactiflora* is a common Chinese medicine used in the treatment of dysentery. Network pharmacology studies have shown that there are 70 common target genes between *Paeonia lactiflora* and ulcerative colitis, involving biological functions such as reaction to drugs, reaction to lipopolysaccharide, positive regulation of nitric oxide biosynthesis, and reaction to estradiol. By acting on the TNF signaling pathway, the cancer signaling pathway, hepatitis B, tuberculosis, and other signaling pathways play a role in the treatment of inflammatory bowel disease and relieve the symptoms of ulcerative colitis (Zhang et al., 2019b).

Schisandrin B, a Chinese herbal component of schisandrin, has been confirmed by network pharmacological studies to alleviate acute colitis by activating AMPK/NRF2-dependent signal transduction ROS-induced mitochondrial damage. It effectively inhibited IL-1β levels and intestinal epithelial cell apoptosis mediated by NLRP3 inflammasome activation in the experimental model of ulcerative colitis (Zhang et al., 2021b). There are many studies on the use of a traditional Chinese medicine in the treatment of ulcerative colitis, indicating that a small number of traditional Chinese medicine in the clinical treatment of intestinal diseases also play a great role. Gallincin, one of the main components of *Quercus infectoria*, has been found to effectively regulate the colonic barrier function and relieve intestinal inflammation in mice. The main mechanism of action may be related to the inhibition of leukocyte infiltration and the reduction of TNF-a, p-ERK1/2, and IL-1β levels (Cui et al., 2020). Coix seed, as one of the functional foods in the Chinese diet, has a significant immunomodulatory effect. Clinical trials have confirmed that Coix seed has the effect of relieving ulcerative colitis. By acting on important targets such as T-cell regulation, the Coix seed feed can maintain the complete blood count at a normal level, relieve oxidative stress, reduce inflammatory cytokine secretion and pathological score, and finally relieve the symptoms of colitis (Zhou et al., 2021b). Gu et al. (2020a) combined network pharmacology technology with GEO gene chips and found that oral administration of Indigo naturalis (IN) could hinder the further development of ulcerative colitis, and the specific therapeutic effect may involve the activation of systemic immunity, mainly acting on the JAK-STAT and IL-17 signaling pathways.

In addition, there have been studies on the treatment of ulcerative colitis, such as Scutellariae radix–Coptidis rhizoma drug pair, which also played an important role in interpreting the mechanism of TCM treatment of ulcerative colitis. As a common combination of Scutellariae radix–Coptidis rhizoma drug pair in the treatment of ulcerative colitis, it was found that it has 43 active ingredients and 134 cross targets, including AKT1, JUN, TNF, VEGFA, and EGFR. The results showed that this drug may play a role in treating diseases through the reaction to toxic substances, positive regulation of cell death, reaction to injury, and reactive oxygen metabolism, involving the P53 signaling pathway, PI3K-Akt signaling pathway, IL-17 signaling pathway, and cancer pathway. The molecular docking results showed that there was a good affinity between the main active ingredients and the core target genes (Niu et al., 2021).

### 4.2 Chinese medicine compound treatment of ulcerative colitis

Studies have shown that 26 active compounds and 148 potential targets in the Guchang Zhixie pill compound related to ulcerative colitis, quercetin, β-sitosterol, and kaempferol are the core active components, while JUN, STAT1, IL-1A, IL-1B, and RELA are the key targets. These targets work by being highly enriched in immune, inflammatory, and oxidative stress–related pathways (Zhang et al., 2021a). Network pharmacologic analysis of Huangtu decoction showed that 47 compounds and 29 targets with the intersection with the disease were significantly enriched in the MMP pathway, which could effectively reduce serum IL-1βand IL-6 levels, and relieve symptoms of ulcerative colitis by inhibiting MMP1, MMP3, and MMP9 (Chen et al., 2021b). Other studies have confirmed that Zuojin pill has 14 active ingredients and 26 key targets based on network pharmacology, through which the active ingredients can directly regulate the toll-like receptor signaling pathway, MAPK signaling pathway, and PI3K-Akt signaling pathway, thus playing a role in the treatment of UC (Wei et al., 2021). The experimental results also show that Zuojin pill is effective in the treatment of chronic atrophic gastritis (Tong et al., 2021). In the Huiyangjiuji decoction on the molecular mechanism of the UC treatment study, the target genes of the validation pathway were found, induced by pathogen infection and tumor-related enrichment in frontal pathways, *in vitro* experiments also confirmed Huiyangjiuji decoction of IL-2, IL-10, and IL-12 interleukins have an obvious inhibitory effect, thus inhibiting the further development of intestinal inflammation (Yu et al., 2021). In other studies, nine active components of Xianglian pill that may have anti-inflammatory effects which were identified through the literature review, and the treatment of ulcerative colitis was mainly through regulating key pathways of the immune system, reducing mucosal inflammation, and achieving anti-inflammatory effects (Liu et al., 2021b).

In addition, a few clinical experiences have led to a prescription for treating ulcerative colitis, the effect is very remarkable, also; it received widespread approbation from people. Xiaokujiedu decoction containing 10 kinds of traditional Chinese medicines was a clinical experience prescription. Through network pharmacology analysis, 103 intersection targets of Dexiaokujiedu decoction and UC were obtained, and 741 biological function enrichment and 124 related pathway enrichment were shown. The five active components of Xiaokuijiedu decoction, namely, quercetin, luteolin, kaoneferol, β-sitosterol, and formononetin, showed good binding activity with core gene targets and played a potential mechanism in treating UC by regulating colonic cell inflammation and oxidative stress–related pathways (Wang et al., 2021b). Quyushengxin formula is also a clinical experience prescription for the treatment of UC, a database of TCM from 41 active ingredients selection, and through the weighted set similarity algorithm to predict the biologically active ingredients of 94 potential protein targets, further functional analysis of these targets and closely related to the progress of biological immunity and inflammation, which play the role of regulating the body’s immune and anti-inflammatory functions (Yang et al., 2019).

Chen et al. (2021a) found that Huai Hua San (HHS) treatment of UC mainly achieves the treatment goal by reducing colon inflammation, while the PI3K/AKT signaling pathway, HIF-1 signaling pathway, and VEGF signaling pathway are identified as the three major signaling pathways related to UC treatment. HHS in the body has been proven to be effective in alleviating UC symptoms. Liu et al. (2021c) also demonstrated that HHS is effective in the clinical treatment of ulcerative colitis, and also believed that the PI3K-Akt signaling pathway may play an irreplaceable role in HHS’s work against UC. Gegen Qinlian decoction for the treatment of abdominal pain and diarrhea contain active ingredients with the highest active target including quercetin, stigmasterol, and kaempferol, and 32 strongly related proteins, including PPARG, AR, RELA, AKT1, and EGFR. Through the significant enrichment in the toll-like receptor signaling pathway, IL-17 signaling pathway, tumor necrosis factor signaling pathway, transcriptional decompression, and other signaling pathways in tumors, the anti-inflammatory, antioxidant, and inhibition of cancer gene transcription can play a role in promoting the intestinal tract to gradually return to normal (Wei et al., 2020). Xu et al. (2021) found that Gegen Qinlian decoction can also regulate immunity, promoting the relief of UC by regulating the immune system. Yu et al. (2020) found through online pharmacological analysis that Banxia Xiexin decoction was involved in biological functions such as drug reaction, oxidative stress response, cell response to lipopolysaccharide, and inflammatory response in the treatment of depression and ulcerative colitis, through the HIF-1 signaling pathway, NF-Kappa B signaling pathway, etc., to achieve the same therapeutic effect for the two different diseases. The example of Banxia Xiexin decoction once again proves that TCM treatment of diseases starts from the pathogenesis. Although the diseases are different with the same pathogenesis, the same treatment method can be adopted to give play to the treatment with TCM characteristics.

As one of the traditional prescriptions for the treatment of colitis, studies have found that the Wumei pill (WMP) has 129 active ingredients and 622 target genes. It exerts anti-inflammatory and antioxidant effects by acting on related signaling pathways such as inflammation, immunity, and tumors, and finally achieves the effect of treating UC (Huang et al., 2021). In another study in UC rats, Ji et al. (2020) found that Sinitang decoction (SNT) could significantly reduce the colonic mucosal injury index and disease activity index. In addition, SNT could reverse the upregulated levels of serum prostaglandin E2 (PGE2), tumor necrosis factor (TNF)–a, nitric oxide (NO), and interleukin (IL)-6 in UC model rats. The classic traditional Chinese medicine formula Jiaoqi powder (JQP) is also one of the potential alternative medicines for UC treatment. Network pharmacology analysis found that the core targets of JQP were mainly enriched in inflammatory and immune response signaling pathways, and *in vivo* experiments in animals showed that JQP ameliorated histological changes and symptoms in DSS colitis by significantly impairing DSS’s ability to induce high expression levels of TNF-α, IL-6, IL-1β, and NF-κB/p65 (Wen et al., 2021). Gan Jiang-Huang Qin-Huang Lian-Ren Shen decoction (GJHQHLRSD) is one of the ancient Chinese traditional recipes, Zhou et al. (2021a) found that the following displayed the strongest combined effects: VEGFA with ginsenoside Rg3, BCL2L1 with diop, ERK1 with worenine, STAT3 with palmidin A, and EGFR with kaempferol, finally confirmed that GJHQHLRSD plays a role in the treatment of UC by regulating the EGFR signaling pathway.

Through database analysis, Jianpi Qingchang Huashi Recipe (JPQCHSR) (Zheng et al., 2021) contains 181 active ingredients and 205 related therapeutic targets, and key compounds include kaempferol, quercetin, and luteolin, these scholars found that JPQCHSR acts on TNF, IL- 17, and other signaling pathways, and ultimately play an important role in repairing intestinal immune damage and reducing the expression of inflammatory factors. It was also found in animal experiments that (Ding et al., 2021) Hudi enteric–coated capsule (HDC) could significantly inhibit the IL-17/JAK2/STAT3 signaling pathway to downregulate the secretion of pro-inflammatory cytokines during the treatment of UC, especially in the middle-dose group. In China, as the only Chinese patent medicine approved for the treatment of UC, five-flavor *Sophora flavescens* enteric–coated capsules (FSEC) are very effective in clinical application. Gu et al. (2020b)found that FSEC can act on the toll-like receptor, TNF, IL-17, NF-kappa B, and other signaling pathways, and finally exert comprehensive therapeutic effects such as anti-inflammatory, immune recognition, and antioxidant (Gu et al., 2020b). Through the aforementioned research, we found that traditional Chinese medicine has a significant effect on the treatment of ulcerative colitis and is widely used in clinical practice. The application of network pharmacology can better demonstrate the specific advantages of traditional Chinese medicine in the treatment of UC, and provide new prospects for the treatment of UC and the wide application of traditional Chinese medicine in the future.

## 5 Discussion

Ulcerative colitis is a common and intractable disease in gastroenterology, and its etiology is not completely clear. Due to the inflammatory nature of UC, improper treatment can lead to further damage to the bowel, increasing the risk of surgery and the development of colorectal cancer (Kirsner, 1979). At present, the incidence and mortality of UC are increasing year by year in the world, and traditional Chinese therapy plays an irreplaceable role in the treatment of UC. In recent years, the development of network pharmacology and the application of the mechanism of action of traditional Chinese medicine treatment of ulcerative colitis to explore a new road, providing a new train of thought, to cure disease-related drugs, all factors such as composition, targets, and pathways into the comprehensive analysis and discussion of it from the whole to consider the idea have the thinking mode of traditional Chinese medicine. By reviewing the mechanism of TCM treatment of ulcerative colitis by using network pharmacology, it is not difficult to see that the characteristics of multiple components, multiple targets, and multiple pathways can be displayed in the treatment of diseases, whether it is a traditional Chinese medicine or a prescription composed of multiple traditional Chinese medicines. In these studies reviewed earlier, the “star target,” “star pathway,” and “star biological process” with high frequency, such as JUN, AKT1, IL-1B, and MAPK signaling pathways, HIF-1 signaling pathway, and PI3K-Akt signaling pathway, regulate immune response, inflammation and other pathways play the role of relieving UC symptoms and thus treating UC. At the same time, while using the network pharmacology method, we have a deeper understanding of the treatment must be based on the original treatment, and the original refers to the pathogenesis, in line with the current development trend of identifying the pathogenesis and treating the disease combined with identifying the disease and treating the disease, for seeking the diagnosis and treatment of disease created a new idea.

Due to network pharmacology being combined with the currently known drugs, composition, the information such as targets, signaling pathways, and biological process to speculate about the action mechanism of the traditional Chinese medicine to treat the disease of a kind of method, can, therefore, comprehensive accurately retrieve the current known information, and the differences caused by the different data collection methods becomes the key to the method. If not handled properly, it will affect the progress of the research. In the aforementioned review, the network pharmacology method was also used to study the treatment of UC by Gegen Qinlian decoction, but the results were not all the same, the reason was that the data collection methods were different between the two types of research. Fortunately, the errors between the two did not affect the further exploration of Gegen Qinlian decoction. In future, while exploring new drug prescriptions, more attention should be paid to animal experiments and cell experiments to verify the prediction results in multi-dimensional and multi-direction, to better guide clinical practice.

What is undeniable is that using the method of network pharmacology of traditional Chinese medicine research, will appear behind the biological information, the latest scientific research achievements in modern pharmacology, and so on, cannot give full play to its ingenuity, has a considerable lag, also artemisinin will not appear again which is such a magnificent great discovery, this has also prompted that we must explore more suitable ways for the TCM research method, to contribute to the development and progress of TCM.
